# Altered Light Sensitivity of Circadian Clock in Shank3^+/–^ Mouse

**DOI:** 10.3389/fnins.2021.604165

**Published:** 2021-02-18

**Authors:** Javier Alamilla, Yazmín Ramiro-Cortés, Adriana Mejía-López, José-Luis Chavez, Dulce Olivia Rivera, Víctor Felipe, Raúl Aguilar-Roblero

**Affiliations:** ^1^Centro Universitario de Investigaciones Biomédicas, Consejo Nacional de Ciencia y Tecnología (CONACYT)-Universidad de Colima, Colima, Mexico; ^2^División de Neurociencias, Instituto de Fisiología Celular, Universidad Nacional Autónoma de México, Mexico

**Keywords:** phase response curve (PRC), ASD, autism, SCN, RHT, NMDA receptor GluN2A, receptor GluN2A, constant light

## Abstract

Autism spectrum disorder (ASD) is a neurodevelopmental disorder characterized by impairment in communication and social interaction, repetitive or stereotypical behaviors, altered sensory perception, and sleep disorders. In general, the causes of ASD remain unknown, but in Phelan–McDermid syndrome, it is known that the disorder is related to the haploinsufficiency of the Shank3 gene. We used an autism model with compromised glutamatergic signaling, the Shank3^+/–^ mouse, to study the circadian rhythm architecture of locomotion behavior and its entrainment to light. We also analyzed the synapse between the retinohypothalamic tract (RHT) and the suprachiasmatic nucleus (SCN), employing tract tracing and immunohistochemical techniques. We found that Shank3^+/–^ mice were not impaired in the SCN circadian clock, as indicated by a lack of differences between groups in the circadian architecture in entrained animals to either long or short photoperiods. Circadian rhythm periodicity (tau) was unaltered between genotypes in constant darkness (DD, dim red light). Similar results were obtained in the re-entrainment to shifts in the light–dark cycle and in the entrainment to a skeleton photoperiod from DD. However, Shank3^+/–^ mice showed larger phase responses to light pulses, both delays and advances, and rhythm disorganization induced by constant bright light. Immunohistochemical analyses indicated no differences in the RHT projection to the SCN or the number of SCN neurons expressing the *N*-methyl-D-aspartate (NMDA) receptor subunit NR2A, whereas the Shank3^+/–^ animals showed decreased c-Fos induction by brief light pulses at CT14, but increased number of vasoactive intestinal polypeptide (VIP)-positive neurons. These results indicate alterations in light sensitivity in Shank3^+/–^ mice. Further studies are necessary to understand the mechanisms involved in such increased light sensitivity, probably involving VIP neurons.

## Introduction

Circadian rhythms are behavioral and physiological processes with a periodicity close to 24h generated by the suprachiasmatic nuclei (SCN), the circadian clock of the hypothalamus (Marcheva et al., 2013). The SCN synchronizes to environmental light *via* a subpopulation of ganglia cells in the retina expressing the photopigment melanopsin (Hattar et al., 2003; Hankins et al., 2008), whose unmyelinated axons form the retinohypothalamic tract (RHT) (Moore and Lenn, 1972). The RHT terminals release glutamate and pituitary adenylate cyclase-activating peptide to SCN ventral neurons (Abrahamson and Moore, 2001; Hannibal, 2002, 2006). Glutamate activates *N*-methyl-D-aspartate (NMDA) and α-amino-3-hydroxyl-5-methyl-4-isoxazole-propionate (AM PA)–kainate postsynaptic receptors (Kim and Dudek, 1991), which increase intracellular calcium in SCN neurons and activate signaling cascades necessary for the synchronization of the circadian clock to light (Ding et al., 1994, 1997).

Autism spectrum disorder (ASD) is a neurodevelopmental disorder characterized by core symptoms of impaired communication and social interaction, repetitive or stereotyped behaviors, and altered sensory perception (Klintwall et al., 2011; Carbonetto, 2014; Baum et al., 2015; Bourgeron, 2015; Robertson and Baron-Cohen, 2017; Schroeder et al., 2017). It is estimated that, worldwide, one in 160 children has ASD (World Health Organization, 2013). The core symptoms are associated with secondary comorbid conditions including intellectual disability, anxiety, gastrointestinal problems, and sleep troubles (Bourgeron, 2015). The causes of ASD remain unknown; however, there are indications of a genetic basis linked to genes of proteins related to synaptic structure and function (Zoghbi and Bear, 2012). One of the best characterized examples is Phelan–McDermid syndrome (PMS), which is caused by a deletion of the Shank3 gene in a single allele in the chromosome 22q13. The main features of PMS are neonatal hypotonia, absent or severely delayed speech, intellectual disability, and autism spectrum disorder (Phelan and McDermid, 2012). The Shank3 gene encodes for the protein SHANK3, a scaffolding protein localized at the postsynaptic of excitatory synapses. SHANK3 scaffold ionotropic and metabotropic glutamate receptors are considered to be key regulators, either directly or indirectly, of synaptic transmission and plasticity (Monteiro and Feng, 2017).

A remarkable feature of ASD is that children manifest a high incidence of sleep problems, of around 53% (Wiggs and Stores, 2004). Interestingly, children with PMS also present an elevated incidence of sleep disturbances, ∼90% of the cases (Bro et al., 2017). Sleep problems and ASD suggest an unexplored link between autism and circadian rhythms. For instance, a circadian oscillation of the SHANK3 in the hippocampus and striatum, which correlates with melatonin serum levels (Sarowar et al., 2016), was recently discovered. Furthermore, it has been reported that knockout mice lacking exon 21 of Shank3 exhibit differences in non-REM sleep and slight differences in free-running circadian rhythms in constant darkness (DD) in comparison to wild-type controls (Ingiosi et al., 2019).

Despite this evidence, the role of the circadian system in autism is unknown. Since SHANK proteins are necessary *via* the PSD-95/GKAP complex, for the organization of NMDA receptors in the postsynaptic density (Lim et al., 1999), and NMDA receptors are crucial for light-induced phase shifts (Colwell and Menaker, 1992; Ding et al., 1994, 1997), we tested whether a compromised glutamatergic signaling in Shank3^+/–^ mice (an animal model linked to autism, with a deletion in the ankyrin repeat domain which encodes for one of the major Shank3 isoforms, Shank3α) affects the circadian rhythm in locomotion, its entrainment to light, the fiber density from the RHT to the SCN, and the number of neurons expressing vasoactive intestinal polypeptide (VIP), NMDA2A subunit, and c-Fos photoinduction in the SCN.

## Materials and Methods

### Animals and Housing

B6(Cg)-Shank3tm1.1Bux/J heterozygous mice (Jax, no. 017889) were bred with C57BL/6J wild-type mice. For aims of housing and breeding but not experimental, the animals were housed under a cycle of 12-h light/dark in a conventional temperature and humidity vivarium, with *ad libitum* access to food and water. Shank3^+/+^ (wild type, WT) and Shank3^+/–^ (HET) littermates including male and female mice were 16weeks old at the beginning of the behavioral experiments. The animals were genotyped at the beginning and again at the end of the experimental procedures to confirm the subject genotype. Data collection and analysis were performed blind with regard to the genotype of the animals until the end of the experiments. All experimental protocols were conducted according to current Mexican legislation NOM-062-ZOO-1999 (SAGARPA), the internal ethical institutional committee guidelines (authorization CICUAL RA-58-15), and the guidelines on the use of animals from the Society for Neuroscience.

We defined DD as the constant exposition to dim red light (5lx) and LD as the exposition to bright light (300 lx, LL) followed by constant dim red light (5 lx).

Before the experiments, the mice were housed for habituation at least for 10 days in groups of four, in the vivarium of the Chronobiology Laboratory, with water and food *ad libitum*. The habituation room was kept at a temperature of 24°C ( ± 1) under a 14:10-h photoperiod (LD 14:10, lights on at 07:00 hours).

### Behavioral Recordings

At the beginning of the experiment, mice were transferred to individual acrylic boxes in cabinets with controlled light–dark daily cycles. Dim red light (5 lx) was constantly present as background throughout the experiment. The circadian rhythm of locomotion was continuously monitored by a computerized system described by Mercado et al. (2009). Briefly, each time the animal moved, electric pulses were generated through pressure sensors placed beneath the floor; these events were computed at 1-min intervals and stored in magnetic media for later analysis. Pairs of animals from both groups (WT and Shank3^+/–^) were monitored simultaneously under the same lighting conditions.

In the first experiment, 22 animals (11 WT and 11 HET) were recorded in different lighting conditions to describe the circadian architecture under long (LD 14:10) and short (LD 10:14) photoperiods. In this experiment, re-entrainment to a 6-h LD advance phase shift was performed. In the second experiment, we analyzed the circadian rhythms under the skeleton photoperiod (SKP) that consisted of two light pulses (1 h, 300 lx) separated by a long (13 h) and a short (9 h) interval of DD (Pittendrigh and Daan, 1976). In SKP, animals are kept in DD for 2 weeks and then two short light pulses per circadian cycle were applied to the DD for at least 21 days. This condition resembles closely the natural conditions of nocturnal animals living in a burrow in the wild. We also analyzed the response to a brief light pulse (15 min, 400 lx) in three characteristic zones (CT06 dead, CT14 delays, and CT22 advances) of the phase response curve (PRC), as previously described for C57BL/6 mice (Schwartz and Zimmerman, 1990).

### Data Analysis

Graphic display of double plots and *χ*^2^ periodogram analysis of the data were made by the Digital Analysis System Applied to Chronobiology (Omnialva/SPAD9) developed and validated in our laboratory (Instituto de Fisiologıìa Celular, Universidad Nacional Autónoma de México). To analyze the architecture of the circadian rhythm, the durations in minutes of activity (*α*), rest (*ρ*), and period (*τ*) were calculated for the different lighting conditions to which the individuals were exposed. For each of the last 10 cycles in each lighting condition, the times of activity onset and offset were recorded. Activity onset was considered as the first point of upward inflection of activity counts in at least five consecutive bins. Activity offset was marked as the first point of downward inflection of activity counts in at least five consecutive bins. The durations of *α*, *ρ*, and *τ* were then calculated for each of the 10 days, with the mean values of these measures being used as individual values for further statistical analysis. For constant lighting conditions, either DD or LL, the period of rhythmicity was estimated from the *χ*^2^ periodogram obtained from the last 10 days of each segment of the experiment. The period of rhythmicity was read from the peak in the range from 15 to 30 h that reached an *α* level of 0.0001; loss of rhythmicity was considered to occur when the amplitude of the peaks did not reach an *α* level of 0.01. Period stability was defined as the inverse of the variance (1/s^2^) from several tau estimates, computed from 10-day intervals, as described in Aschoff (1984). We also computed the days it took to synchronize the animals to 6-h advances in the LD cycle. We considered that the subject was entrained when the duration of *α* was similar to the mean value before the LD shift.

To analyze the circadian rhythms under the SKP and the PRC, the recordings were conducted in dim red light (5 lx) for at least 10 basal days. In nocturnal rodents, circadian time 12 (CT12) is designated as the beginning of the subjective night indicated by the onset of the active phase. CT12 was estimated by adjusting a line to the onset of activity of at least 10 cycles; *τ* was then calculated from the slope of this line. To analyze the characteristic zones of the PRC, on day 11, a 15-min light pulse was applied either at CT06, CT14, or CT22, each one projected from CT12 on day 10, and the recordings continued in DD for at least 14 days. The phase shifts in the activity onset were estimated on the day of the light pulse by comparing the line adjusted to CT12 from the last 10 days of recording projected to the day the light pulse was applied with the corresponding line adjusted before the light pulse. To analyze the circadian rhythms under SKP, on day 11, the recordings continue for at least 21 days under a DD background on which two 1-h light pulses are separated by long (13 h) and short (9 h) DD intervals.

### RHT Tracing

After the behavioral recordings were finished, subsets of animals from both groups were anesthetized with fluothane vapors. Cholera toxin β-subunit (CTB, List Biological) was injected into the vitreous of one eye (1.5 μl of 0.2% CTB in 2% dimethyl sulfoxide/0.9% saline solution at a rate of about 0.3 μl/min). Forty-eight hours later, the animals were further processed for immunohistochemical staining, as described below.

### c-Fos Photoinduction

In order to establish the SCN response to brief light pulses, different subsets of animals (from both groups) were recorded in DD for at least 7 days and CT12 was determined as previously described. A 15-min light pulse was applied the next day at CT14 projected from CT12. Another set of WT animals received a 15-min light pulse at CT06 and were used as baseline to compare the c-Fos photoinduction at CT14. Both sets of animals were contrasted with animals manipulated at the same circadian times (CTs) but did not receive light pulses. All animals were further processed for immunohistochemistry, as described next.

### Histological Procedures

At the conclusion of the previous experiments, the animals received a lethal dose of pentobarbital sodium (60 mg/kg body weight) and were transcardially perfused with 50 ml of 0.9% saline solution followed by 100 ml of paraformaldehyde–lysine–periodate fixative, consisting of a solution of 4% paraformaldehyde in phosphate-buffered saline (PBS; 0.1 M, pH 7.2) added with lysine (75 mM) and *m*-periodate (10 mM). Brains were dissected and post-fixed for 1 h at 4°C with paraformaldehyde–lysine–periodate and then cryoprotected by successively immersing the brain in 10%, 20%, and 30% sucrose solutions. Coronal sections 30 μm thick were obtained using a cryostat at −23°C. All sections obtained throughout the SCN were collected in three sets in ice-cold PBS (0.1 M, 0.15 M NaCl, pH 7.2), then processed for immunohistochemistry according to the avidin–biotin method. All the primary antibodies used in this research have been previously used and tested. Primary antibodies were diluted as indicated in 0.1 M PBS containing 1.0% normal serum in 0.3% Triton X-100 [anti-VIP raised in rabbit, CAT 20077, Incstar, 1:2,000 (Patton et al., 2020); anti-CTB subunit raised in goat, CAT 703, List Biological Laboratories, 1:2,000 (Zhao et al., 2015); anti-NMDAR2A (GluN2A) raised in rabbit, CAT ACG 002, Alomone Labs, 1:400 (Atkin et al., 2015); and anti c-FOS raised in rabbit, CAT SC-52, Santa Cruz, 1:1,000 (Li and Spitzer, 2020)]. Sections from each bin were incubated with one of the primary antibodies for 72 h at 4°C, then rinsed in PBS and incubated for 2 h at room temperature in biotinylated secondary antibodies (goat anti-rabbit, rabbit anti-goat, and goat anti-rabbit immunoglobulin G), respectively, rinsed in PBS, and incubated for 2 h at room temperature in avidin–biotin–peroxidase complex, rinsed again in PBS, and pre-incubated for 5 min in 0.05% diaminobenzidine (DAB) in Tris buffer (pH 7.2). Of 30% hydrogen peroxide, 35 μl was then added to each set of sections and left to react for an additional 5 min. These procedures were done simultaneously in control and experimental tissues. Secondary antibodies (Alexa Fluor 488: donkey anti-goat and goat anti-rabbit, both from Abcam, Cambridge, UK) were used at a 1:200 dilution. Sections were then mounted with PBS (pH 7.2), air dehydrated, and covered with Permount (Fisher Scientific, Hampton, NH) and stored until inspected by microscopy. In an additional set of sections, the primary antibody was omitted as a control for nonspecific binding of the secondary antibody (Supplementary Figure 1).

### Image Acquisition

SCN slices stained with DAB were visualized in an Olympus BX51 microscope (Shinjuku City, Tokyo, Japan), whereas immunofluorescence staining was imaged in two different confocal microscopes: a TCS-SP5 II (Leica, Heidelberg, Germany) and a LSM710 (Carl Zeiss, Oberkochen, Germany). Only complete and undamaged sets of sections were used for imaging analysis. The software used for image acquisition were Image-Pro Plus v.4.1 (Media Cybernetics, Silver Spring, United States) and Zen 12 (Carl Zeiss). A panoramic view was obtained with a ×10 objective. Subsequently, ×20 and ×40 were used to analyze SCN neuronal populations by stereology.

For light microscopy, steps of 10 μm were employed to capture at least three micrographs from each section, with at least three sections used from each brain. For confocal microscopy, steps of 1 μm were employed to capture optical slices (around 20 optical slices were gathered per image). A minimum of three SCN sections were acquired for each brain. Parameters such as laser intensity, resolution, gain, and digital offset were adjusted with the WT group, taking care to ensure there was no saturation and the parameter adjustments were maintained constant for subsequent images across both genotypes.

### Image Analysis

SCN neurons immunolabeled for c-Fos, VIP, NMDAR2A, and CTB-labeled RHT fibers were counted by stereology using the optical fractionator method of Gundersen (Mouton, 2011) according to:

TotalN=ΣQ*(F)1*(F)2*(F)3

where Total *N* is the total neuronal number, Σ*Q* is the number of objects actually counted, *F*_1_ = 1/(number of sections analyzed/total number of sections), *F*_2_ = 1/(area of the dissector frame/area of the *x*–*y* step), and *F*_3_ = 1/(dissector height/section thickness). The SCN on one side of the brain was photographed by light or confocal microscopy, as described above. Using ImageJ software (1.53c), the numbers of neuronal somata within an unbiased optical dissector (volume = 105 μm^3^) were counted from photographs of three out of 11–12 SCN sections (which comprise its rostrocaudal extent). The dissectors were distributed at an *x*–*y* step of 120 μm with the aid of a Grid tool from ImageJ. For the CTB-labeled RHT fibers, a cycloid grid from ImageJ was used as the unbiased dissector.

### Statistical Analysis

The data from the circadian architecture are given as the mean ± standard error of the mean (SEM). Latency to entrain to phase shifts, SKP, and the labeled RHT fibers are shown as box and whisker (5–95 percentiles) bars. Characteristic PRC regions are shown as the mean and SEM. Normal distribution (Shapiro–Wilk) and the homogeneity of variances (Levene test) were evaluated before the use of parametric statistics. To analyze differences between the experimental groups, Students *t* test for independent samples was used. Statistical analyses for c-Fos were performed with a three-way ANOVA with Tukey’s *post hoc*. All statistical analyses were done using GraphPad Prism 7.0 (GraphPad Software, La Jolla, CA, United States). The *α* level was set at 0.05.

## Results

### Behavior

**FIGURE 1:**
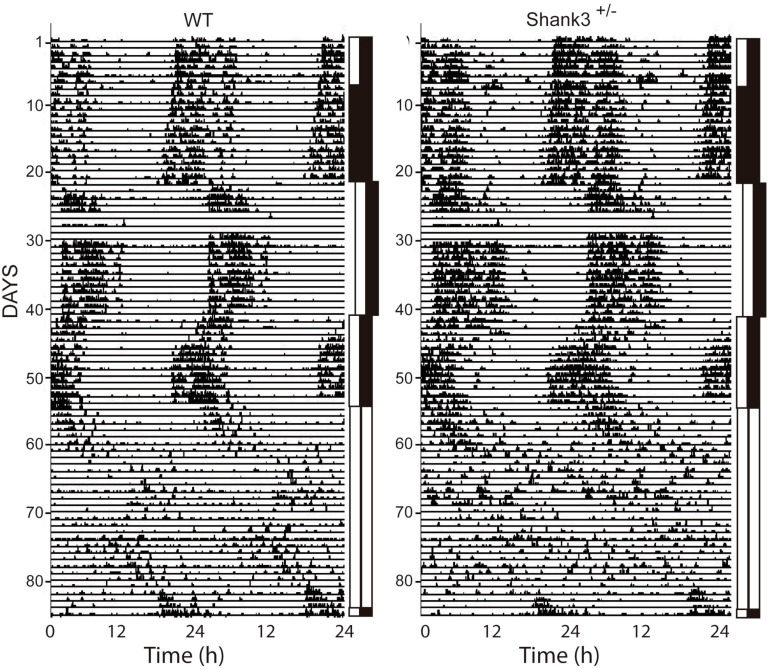
Representative examples of double-plot actograms from wild-type (WT) and Shank3^+/–^ mice. The different lighting conditions are indicated as *vertical bars beside each actogram*. The LD condition is represented by *black* and *white bars*; DD, *double black bars*; and LL, *double white bars*. In these actograms, animals were submitted to a long photoperiod (14:10). The *shift to the right in the bars*, approx. recording days 20–40, indicates a 6-h delay in lights-on time. The 6-h advance is shown as a shift back in the lights-off time. The days of recording are on the *left* and the time of recording (in hours) at the *bottom*.

**FIGURE 2:**
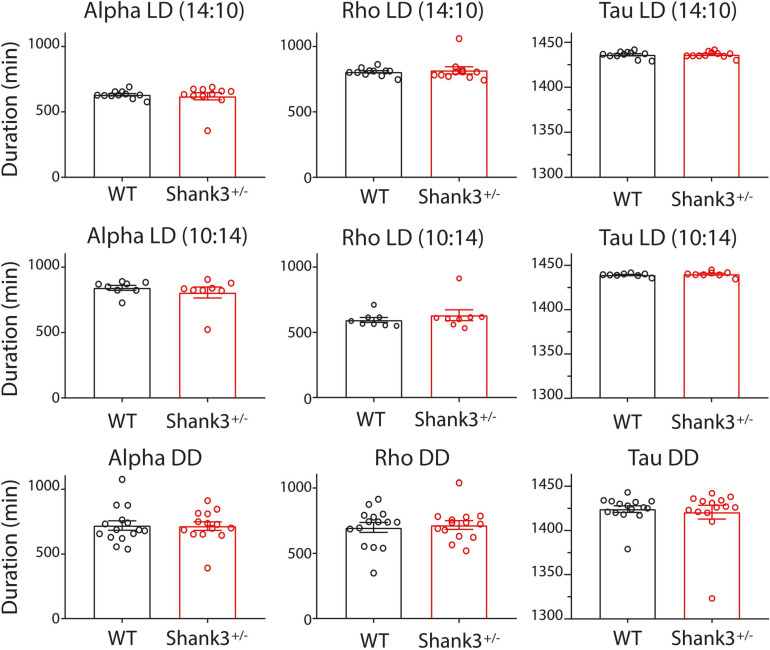
Architecture of circadian rhythms for wild type (WT) and Shank^+/–^ under different lighting schedules. Bar graphs (mean ± SEM) and individual dots of the circadian rhythm architecture from WT (*black*) and Shank3^+/–^ (*red*). The *columns* indicate (from *left to right*) the durations (in minutes) of activity (*α*), rest (*ρ*), and period (*τ*) estimated from 10 days of recording. The *rows* indicate the lighting schedules (from *top to bottom*): long photoperiod, short photoperiod, and DD (constant dim red light).

Examples of double-plot actograms under different lighting conditions from the first experiment are shown in Figure 1; the left and right panels are representative examples for WT and Shank3^+/–^. No significant differences were found in the circadian architecture of WT and Shank3^+/–^ mice under different lighting conditions (Figure 2).

Under the long photoperiod (LD 14:10), the durations of activity (*α*), rest (*ρ*), and period under entrained conditions (*τ*^∗^) for WT were (mean ± SEM in minutes, for this and subsequent photoperiods): 631 ± 9, 806 ± 9, and 1,436 ± 1, respectively, and for Shank3^+/–^ were 619 ± 28, 818 ± 28, and 1,436 ± 1, respectively (Figure 2, top). The inter-individual period stability was lower in WT with respect to Shank3^+/–^ (0.07 and 0.1, respectively).

As expected, under the short photoperiod (LD 10:14), the *α*, *ρ*, and *τ* for WT were (in minutes) 842 ± 19, 595 ± 19, and 1,437 ± 1, respectively; in Shank3^+/–^, *α*, *ρ*, and *τ* were 806 ± 42, 632 ± 42, and 1,438 ± 1, respectively (Figure 2, center, and Supplementary Figure 2). Period stability under the short photoperiod decreased from 0.36 in WT to 0.13 in Shank3^+/–^.

As *α*, *ρ*, and *τ* were not significantly different between WT and Shank3^+/–^ animals, in the long (14:10) and short (10:14) photoperiods, therefore, we did not further evaluate such parameters in a regular 12:12 photoperiod.

Additional phase angles of activity (*ψα*) and rest (*ψρ*) were evaluated for the long and short photoperiods in WT and Shank3^+/–^ animals. No significant differences were found in *ψα* and *ψρ* between the different groups of animals at the different photoperiods analyzed (Table 1).

**TABLE 1:** Phase angle of activity (α) and rest (ρ) for animals maintained in two different photoperiods.

Photoperiod	ψα			ψρ		
	WT	Shank3^+/–^		WT	Shank3^+/–^	
14:10	5.5 ± 3.2	0.3 ± 6.1	t=0.75, df=14, p=0.5	81.6 ± 22.0	23.8 ± 38.0	t=1.3, df=14, p=0.2
10:14	12 ± 2.1	7.6 ± 5.4	t=0.75, df=14, p=0.5	93.0 ± 20.0	59.0 ± 48.8	t=0.63, df=14, p=0.5

*No significant differences between groups were found. Positive values indicate that α or ρ, begin before light was off or on, respectively.*

**FIGURE 3:**
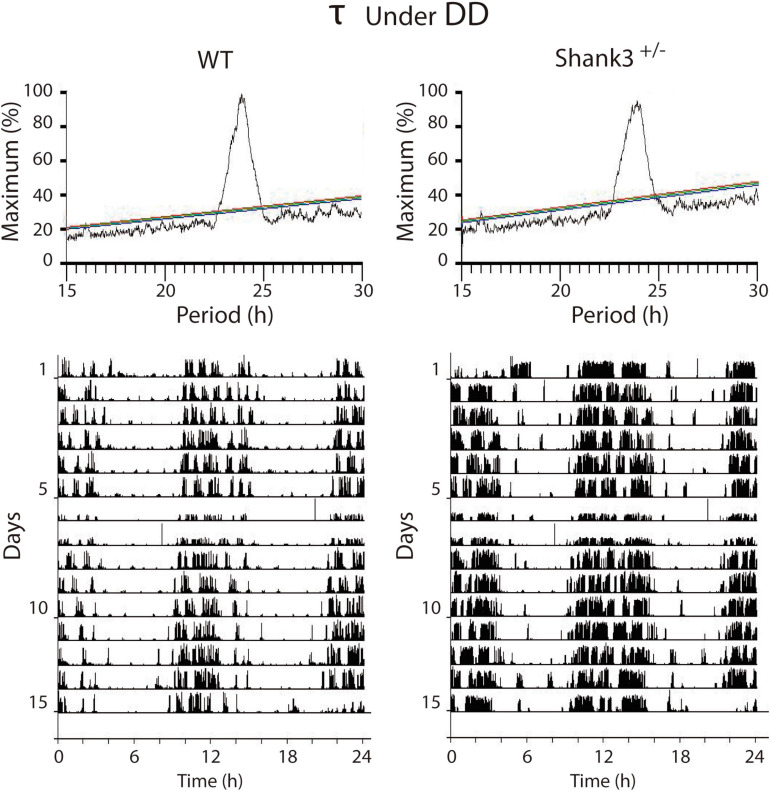
Representative examples of the *χ*^2^ periodograms from wild-type (WT) and Shank3^+/–^ mice in DD. There were no differences between groups in the endogenous period. The actograms at the *bottom* show the data segments used to calculate the *χ*^2^ periodogram. QP is normalized as a percentage of the maximal peak value. For Figures 3, 4: *different color diagonal lines* indicate statistical significance (*p* < 0.05, 0.01, 0.001).

In DD, the *α*, *ρ*, and *τ* for WT were, respectively 719.7 ± 36.7; 698.5 ± 37.9, and 1424 ± 3.7, while those for Shank3^+/–^ were 715.6 ± 32.9, 716.9 ± 33.1, and 1421 ± 7.9, respectively (Figure 2, bottom). The period stability in DD was higher in WT with respect to Shank3^+/–^ (0.005 and 0.001, respectively). The *χ*^2^ periodogram for all days in DD showed one peak, 1,428.0 ± 6.1 and 1,438.2 ± 1.2 for each group, respectively. Representative *χ*^2^ periodograms under DD from each group are shown in Figure 3.

**FIGURE 4:**
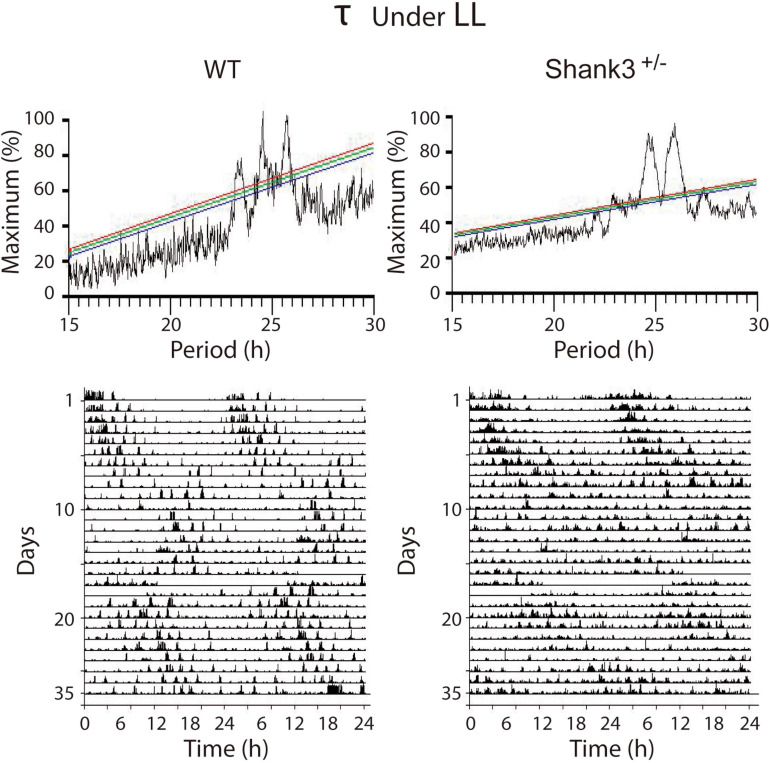
Representative examples of the *χ*^2^ periodograms from wild-type (WT) and Shank3^+/–^ mice in LL. Constant light induces a clear disruption of the architecture of circadian rhythms in both groups characterized for showing at least two peaks in the QP values. The actograms at the *bottom* show the data segments used to calculate the *χ*^2^ periodogram. The QP amplitude is normalized as a percentage of the maximal peak value.

Under LL, both groups of animals showed an initial increase in *τ* to 1,482.1 ± 12.0 h for WT and 1,506.2 ± 18.1 h for Shank3^+/–^; after ∼6 days, an additional increase to 1,536 ± 2.4 for WT and 1,590.2 ± 48.3 for Shank3^+/–^ was observed. After ∼13 days in LL, one WT (1/12, 8.3%) and all Shank3^+/–^ mice (11/11, 100%) were behaviorally arrhythmic (*p* < 0.0001, Fisher’s exact test) (Supplementary Figure 3). Overall, the *χ*^2^ periodogram for all days recorded under LL showed that both groups showed two main peaks (*τ*), 1,471.8 ± 6.1 and 1,531.8 ± 7.2 for WT and 1,471.2 ± 6.6 and 1,578.1 ± 5.4 for Shank3^+/–^. A statistical difference was found when we only compared the longer *τ* between groups (*t* = 5.2, *p* = 0.0002, unpaired *t* test). Additional minor peaks were found in some animals from both groups. In WT animals, a third peak was found in 50% of the cases, whereas in Shank3^+/–^ mice a similar phenomenon was detected in 30% (Figure 4).

**FIGURE 5:**
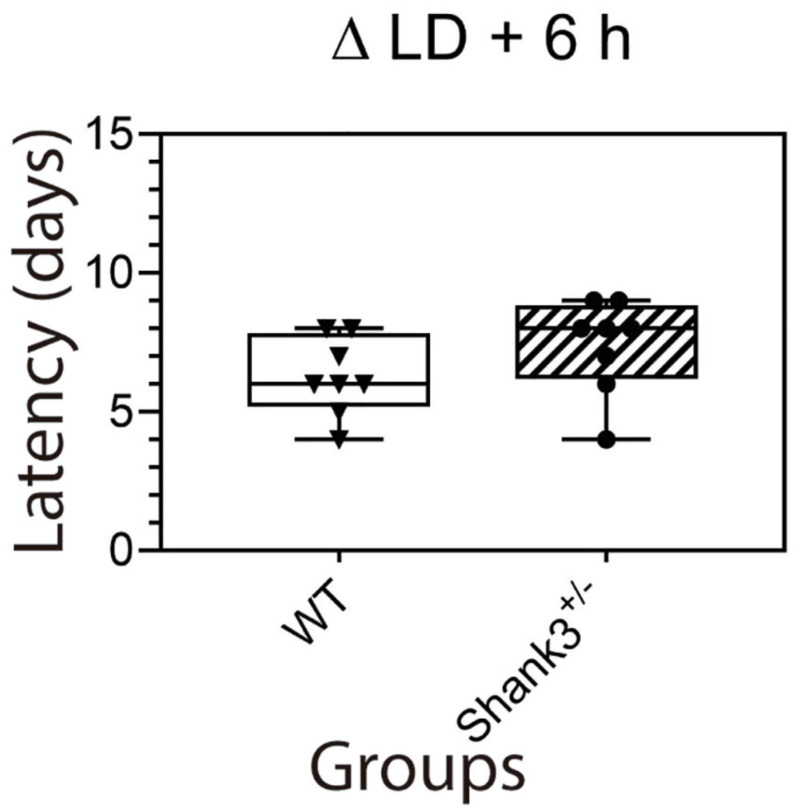
Latency to re-entrain to LD 6-h shifts. There were no differences between groups in the number of days to re-entrain to a 6-h advance in the lights-on time. Box and whiskers (5–95 percentiles) from wild type (WT, *open box*) and Shank3^+/–^ (*hatched box*). Individual data are also plotted.

Under the long photoperiod, the latency (in days) to entrain to LD advances were, for WT and Shank3^+/–^ (median ± minimum–maximum), 6 ± 4–8 and 8 ± 4–9, respectively (Figure 5 and Supplementary Figure 4).

**FIGURE 6:**
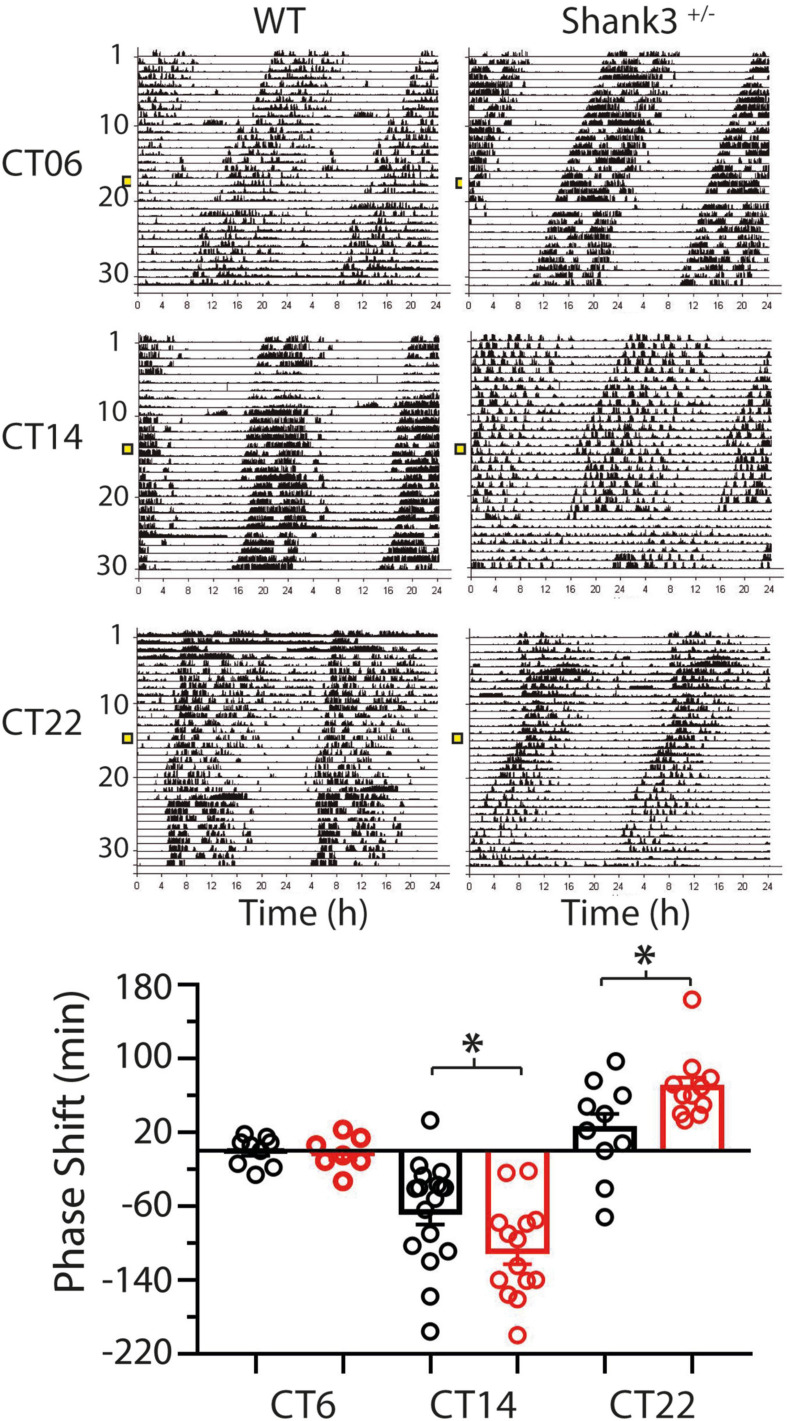
Differences in the phase shifts at two characteristic times of the phase response curve (PRC) between wild-type (WT) and Shank3^+/–^ mice. Fifteen-minute light pulses of white light (400 lx) given to mice kept in DD induce either a delay or an advance in the onset of activity depending on the time of presentation (CT14 or CT22, respectively), except at CT6 when no phase response was induced. In all cases, Shank3^+/–^ (*red bars*) show larger responses than WT (*black bars*) mice. Positive values correspond to phase advances and negative values correspond to delays. Mean ± SEM in minutes are plotted. ^∗^*p* < 0.05, *t* test with Welch’s correction.

Remarkably, significant differences between WT and Shank3^+/–^ animals were found in the PRC (Figure 6). Light pulses applied at CT6 either in WT (−0.1 ± 5.2 min) or Shank3^+/–^ (−2.4 ± 7 min) had no effect on the phase of the rhythm [*t*_(12)_ = 0.27, *p* = 0.79, unpaired *t* test with Welch’s correction]. At CT14, phase delays were −66 ± 14 min in WT and −109 ± 14 min in Shank3^+/–^ [*t*_(29)_ = 2.2, *p* = 0.035, unpaired *t* test with Welch’s correction]. At CT22, phase advances were 23.8 ± 16 min for WT and 69 ± 11 min for Shank3^+/–^ [*t*_(16)_ = 2.72, *p* = 0.037, unpaired *t* test with Welch’s correction].

### Histology

**FIGURE 7:**
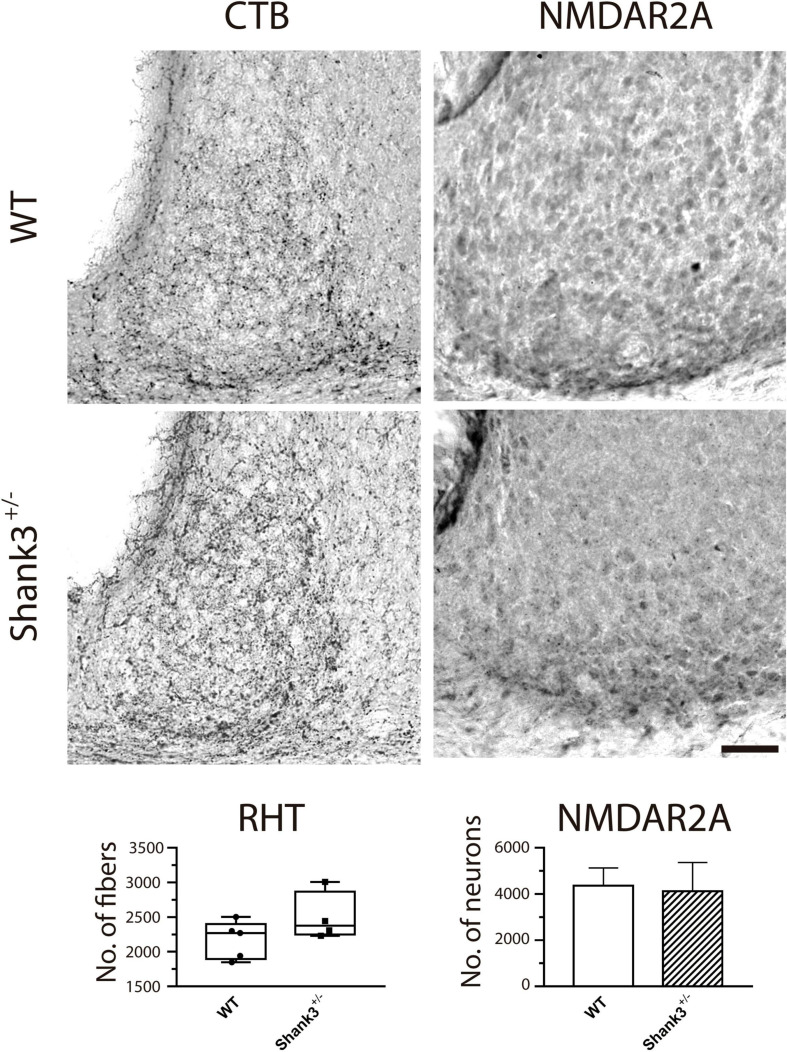
Immunostaining for cholera toxin β-subunit (CTB) and NMDAR2A in the suprachiasmatic nucleus (SCN) from wild-type (WT) and Shank3^+/–^ mice. CTB (diaminobenzidine, DAB, *left column*) and NMDAR2A (*right column*) did not show obvious differences in the immunoreactivity between groups (WT, *top micrographs*; Shank3^+/–^, *bottom micrographs*). For this and subsequent figures (Figures 8, 9), *scale bar* = 50 μm.

Figure 7 shows representative examples of immunostaining to CTB and the NMDAR2A. Our results indicated no important differences in the number of retinal axons impinging on SCN neurons using DAB (Figure 7, left panels) and the fluorescence expression coming from such terminals [96.8 ± 38.4 and 130.5 ± 42.7 AU × mm^2^ for WT and Shank3^+/–^, respectively, six animals in each group (data not shown)]. Similarly, no differences were found in the amount of SCN neurons expressing the NMDAR2A subunit (Figure 7, bottom left panel).

**FIGURE 8:**
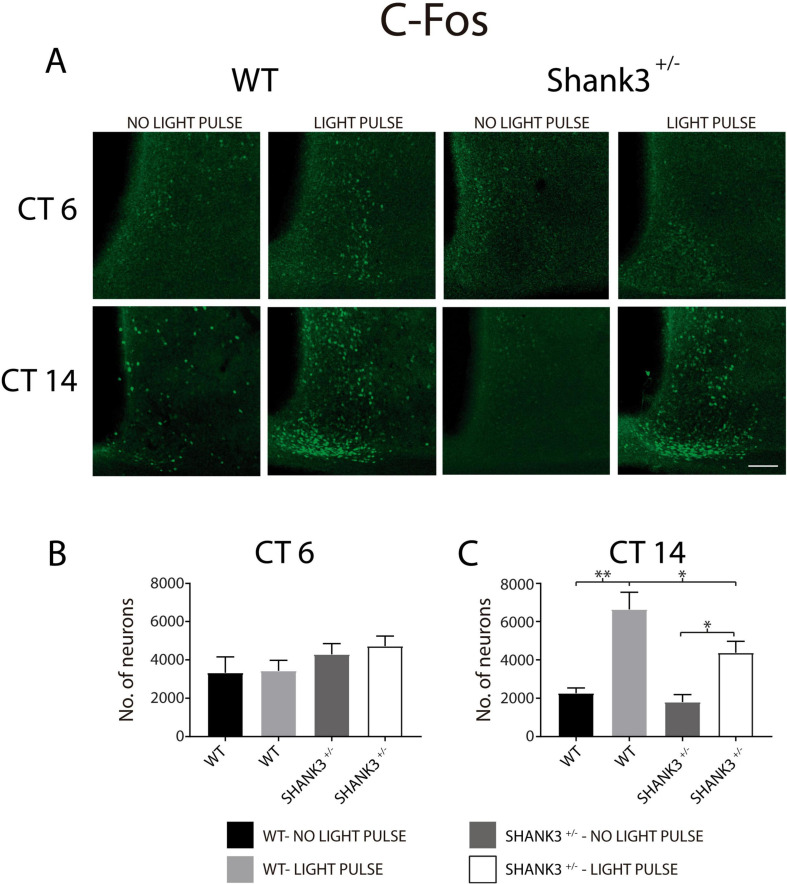
c-Fos immunoreactivity of suprachiasmatic nucleus (SCN) neurons in wild-type (WT) and Shank3^+/–^ mice. **(A)** Micrographs of c-Fos immunostaining in WT and Shank3^+/–^ animals, with and without light pulse, and analyzed in two circadian times (6 and 14). Graphs depict the number of SCN neurons immunopositive to c-Fos at CT6 **(B)** and CT 14 **(C)**. For (**C**): ^∗^*p* < 0.05, ^∗∗^*p* < 0.01, Tukey’s multiple comparisons test.

On the other hand, the expressions of c-Fos and VIP were analyzed in WT and Shank3^+/–^ animals. Immunopositive SCN neurons to c-Fos were counted in WT and Shank3^+/–^ animals, at two times of assessment (CT6 and CT14) and in the presence or absence of the light pulse (Figure 8A). Analyses performed with three-way ANOVA on the data (Table 2) indicated a significant interaction as a result of the CT of experimentation [*F*_(1, 26)_ = 13.2, *p* = 0.001]. Also, the CT of experimentation and the genotypes of the animals showed a relevant interaction [*F*_(__1, 26)_ = 5.8, *p* = 0.02]. Finally, the CT of experimentation and the presence or absence of the light pulse had a significant interaction [*F*_(__1, 26)_ = 9.8, *p* = 0.004]. It is worth noticing that at CT6, the numbers of SCN neurons with c-Fos immunostaining were not different in both genotypes of animals with and without the light pulse (Figure 8B). However, at CT14, WT animals showed higher numbers of c-Fos-immunopositive neurons induced by the light pulse in comparison to the Shank3^+/–^ mice that received the light pulse. The animals that did not receive the light pulse showed significantly lower numbers of ventrolateral SCN neurons in comparison to their counterparts that received the light pulse (Figure 8C).

**TABLE 2:** SCN neurons expressing C-Fos immunoreactivity in WT and Shank3^+/–^ animals (*n*), at two CT of experimentation (CT6, CT14).

	WT-No light pulse	*n*	WT-Light pulse	*n*	Shank3^+/–^ No light pulse	*n*	Shank3^+/–^ Light pulse	*n*
CT6	3333 ± 827	7	3428 ± 549	4	4293 ± 557	3	4720 ± 525	3
CT14	2260 ± 278	4	6653 ± 891^#^	4	1801 ± 389*	4	4378 ± 593*	5

Tukey’s multiple comparisons test: WT (#) *p* < 0.01 presence vs. absence of light pulse; Shank3+/- (*) *p* < 0.05 presence *vs.* absence of light pulse.

**FIGURE 9:**
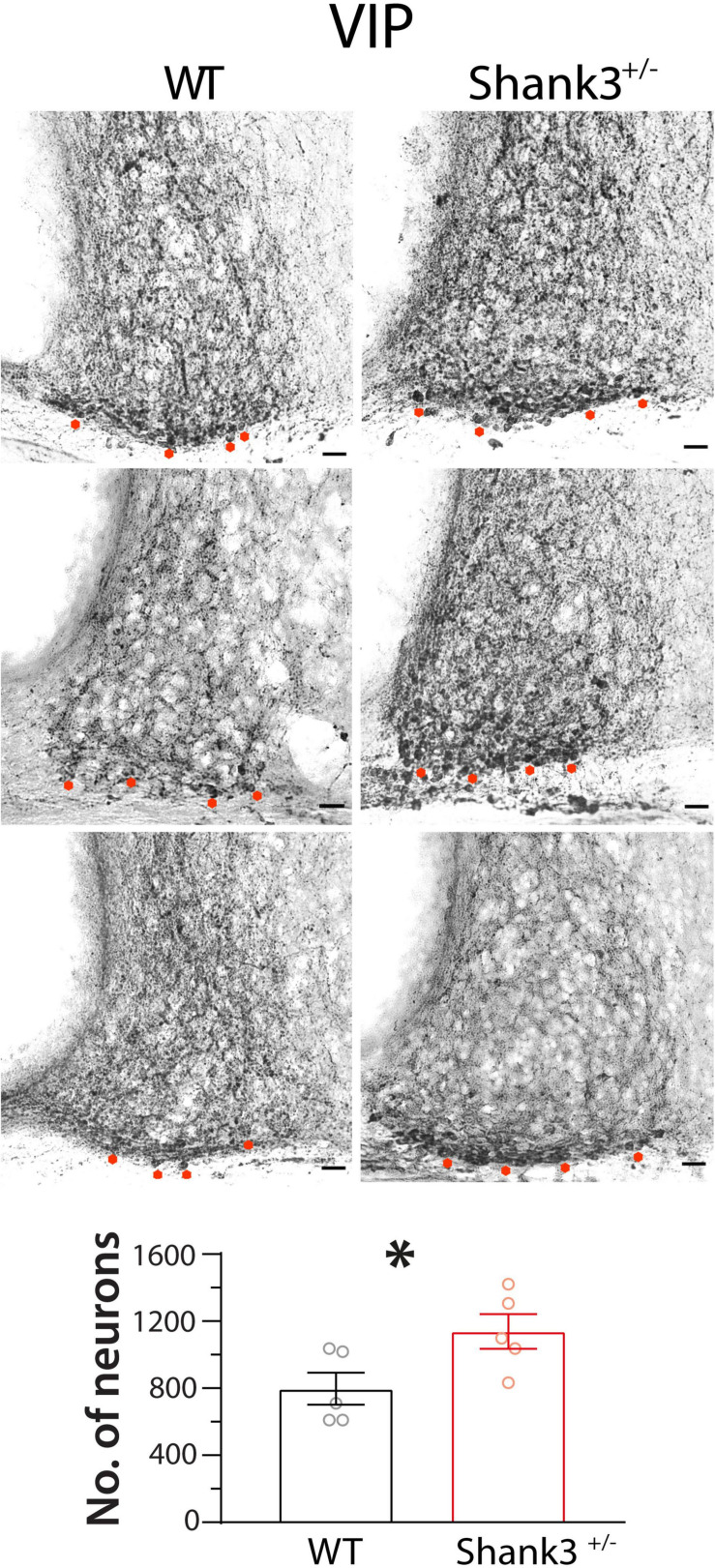
Vasoactive intestinal polypeptide (VIP) immunostaining in suprachiasmatic nucleus (SCN) neurons of wild-type (WT) and Shank3^+/–^ mice. Shank3^+/–^ animals (*right column*) showed higher numbers of VIP-immunopositive SCN neurons than WT mice (*left column*, five animals in each group). *Asterisks* indicate SCN neurons immunopositive to VIP. ^∗^*p* < 0.05, unpaired *t* test.

Regarding VIP expression, we found higher numbers of VIP-immunopositive neurons in Shank3^+/–^ animals (1,423 ± 129) in relation to WT mice [996 ± 120; *t*_(8)_ = 2.4, *p* = 0.04, unpaired *t* test] (Figure 9).

## Discussion

At the behavioral level, here, we report that Shank3^+/–^ mice did not show modifications in the circadian rhythm functioning, implying no alteration in the SCN physiology, as indicated by the lack of differences between groups in the circadian architecture: the tau was unaltered in DD, the durations of alpha and rho were similar between genotypes in entrained animals to either the long or short photoperiod, the re-entrainment to shifts in the L/D cycle, and in the SKP. However, we did find differences in the response to light between groups, as indicated by the larger phase responses induced by 15-min bright light pulses, both delays and advances, and the differences in rhythm dynamics (progress to overt arrhythmic pattern) induced by constant bright light. Moreover, our histological analysis indicated changes in WT and Shank3^+/–^ in c-Fos expression and in the amount of VIP-immunoreactive SCN neurons. These differences indicate alterations in light sensitivity in Shank3^+/–^ mice with respect to their WT siblings.

It is worth noting that Shank3^+/–^ mice did not show changes in the duration of the activity/resting phase and the period of the circadian rhythm of locomotion, in the short and long photoperiods, as well as the time taken to reach synchronization to a 6-h shift. Moreover, Shank3^+/–^ mice did not present any disturbance in the above-mentioned parameters when they were under DD, which is consistent with the data reported for SHANK3^ΔC^ mice, which do not show a disruption in the circadian rhythms specifically in alpha or period length during the DD period (Ingiosi et al., 2019). Nevertheless, in that study, the authors described reduced wheel-running activity as well as differences in the architecture of sleep in SHANK3^ΔC^ animals. We think that these contrasting results in relation to our study may be due to our use of heterozygous mice instead of knockout mice. In addition, the differences can be related to the fact that the wheel-running activity is considered a self-motivated rewarding behavior (Janik and Mrosovsky, 1993; Marchant and Mistlberger, 1996; Pendergast et al., 2014) that can differ from the parameters of the circadian rhythm and its synchronization to light analyzed in the present research.

We found no evident differences between Shank3^+/–^ and WT mice in the immunohistochemical staining of the RHT projections and the NMDA receptor subunit 2A in SCN neurons. However, Shank3^+/–^ animals showed a decreased c-Fos photoinduction at CT14 in SCN neurons, whereas VIP-positive neurons were augmented. c-Fos expression is used to detect neuronal trans-synaptic activation (Morgan and Curran, 1991). Therefore, it is tempting to consider that a lower c-Fos immunoreactivity in Shank3^+/–^ animals after a light pulse may indicate reduced glutamatergic signaling in some point of the RHT–SCN pathway. Moreover, an increase in SCN VIP neurons, which are essential for the light-mediated resetting (Jones et al., 2018; Mazuski et al., 2018), may be the result of a compensatory mechanism.

Since SHANK3 is a scaffold protein for AMPA, NMDA, and the metabotropic glutamate receptors in the postsynaptic density (Sheng and Kim, 2000), the reduced c-Fos expression in Shank3^+/–^ SCN neurons may be an expected outcome. Nevertheless, it is likely that corrective mechanisms take place in the Shank3^+/–^ circadian system to maintain constant synaptic functions, such as the increase in VIP neurons in Shank3^+/–^ animals. Some studies manipulating the Shank3 gene show an increase in the frequency of miniature glutamatergic events in the Schaffer collateral-CA1 in the hippocampus (Bozdagi et al., 2010). Other studies show that spine length is increased at 4 weeks of age but decreased at 10 weeks in dendrites of hippocampal CA1 neurons, but no differences in miniature inhibitory postsynaptic potentials were found (Wang et al., 2011). Still other studies show a decrease in the amplitude of miniature glutamatergic events in cortico-striatal connections (Peça et al., 2011; Zhou et al., 2016). A plausible explanation for our results is that the higher behavioral response of the Shank3^+/–^ mice to brief light pulses in DD at CT14 and CT22 is the result of an augmented sensibility to light related to the augmented expression of VIP-positive neurons in the SCN that might have resulted from a compensatory mechanism of an impairment in the retinal communication to the SCN.

The period stability index found in WT and Shank3^+/–^ mice in different photoperiods is interesting. Our results suggest that Shank3^+/–^ animals had more stable periods in long photoperiods (14:10), whereas this situation was reversed in short photoperiods (10:14). However, it is worth noting that the stability found in Shank3^+/–^ mice is quite similar in both photoperiods (0.1 and 0.13, long and short, respectively), but in WT animals, the short photoperiod shows higher variability (0.07 and 0.36, long and short photoperiods, respectively). Perhaps the higher stability found in the periods of Shank3^+/–^ mice, along different photoperiods, is a result of a greater responsivity to light, which may be related to the result that Shank3^+/–^ mice showed an increased response to light in the PRC.

It should be mentioned that even though the constant dim red light (CDRL) exposition is a broadly used experimental approach to solve maintenance and experimental procedures, it is not a perfect equivalent alternative to complete darkness; given that, CDRL has physiological effects on the circadian rhythms in comparison to DD (Gonzalez, 2018). However, we consider unlikely that the exposure of Shank3^+/–^ mice to CDRL instead of DD can hide modifications in circadian rhythms because both groups of animals were exposed to similar light environmental conditions in concurrent times.

The increased light sensitivity in Shank3^+/–^ could also be related to the increased latency of the pupillary light reflex reported in children with ASD and in the Shank3 macaque (Daluwatte et al., 2013; Zhou et al., 2019). Furthermore, several lines of evidence indicate high levels of glutamate in serum and brain structures (using proton magnetic resonance spectroscopy) in children diagnosed with autism (Rojas, 2014). The decreased c-Fos photoinduction at CT14 found in Shank3^+/–^ SCN neurons is contradictory to the overreaction of Shank3^+/–^ mice to the light pulse in the PRC, which may indicate that c-Fos photoinduction is not directly related to the direction (either advance or delay) of the behavioral phase shifts. This could be related also to the common sleep problems shown by children with this developmental pathology (Wiggs and Stores, 2004; Bro et al., 2017; Veatch et al., 2017), which consist of long sleep latencies and delayed or advanced sleep onset or offset. It is likely that a small amount of light is sufficient to synchronize children with autism to a new schedule, whereas a similar amount of light is impotent to synchronize a regular person.

A caveat of the present research is that we did not explore sex differences in this research. Autism spectrum disorder has a higher prevalence in males than females in a magnitude of 2:1–3:1 (Halladay et al., 2015). Moreover, it is likely that the existence of a sexual dimorphism in the circadian rhythms of autism models, such as the Shank3^+/–^ mice, awaits being addressed.

## Conclusion

In conclusion, Shank3^+/–^ mice showed a higher response in the PRC than their wild-type littermates. However, no changes were evident in the general architecture of the circadian rhythms. Histological analyses indicated a decrease in c-Fos photoinduction in Shank3^+/–^ SCN neurons at CT14, whereas augmented VIP-positive neurons were found in such animals. In this regard, we hypothesize that the circadian system of Shank3^+/–^ mice compensates the impairment of the RHT–SCN communication with an overexpression of VIP SCN neurons, which may result in larger phase shifts induced by light. More research is necessary to understand the cellular processes that affect synchronization in Shank3^+/–^ animals, which may shed light on the problems related to circadian rhythm in patients diagnosed with this developmental pathology.

## Data Availability Statement

The raw data supporting the conclusions of this article will be made available by the authors, without undue reservation.

## Ethics Statement

The animal study was reviewed and approved by Internal ethical committee from the Instituto de Fisiología Celular de la Universidad Nacional Autónoma de México (authorization CICUAL RA-58-15) in accordance to the guidelines on the use of animals from the Society for Neuroscience.

## Author Contributions

JA, YR-C, and RA-R contributed to generate the hypothesis, provide animals and materials, design the study, analyze the data, and wrote the manuscript. AM-L, J-LC, DR, and VF conducted the experiments and analyze the data. All authors contributed to the article and approved the submitted version.

## Conflict of Interest

The authors declare that the research was conducted in the absence of any commercial or financial relationships that could be construed as a potential conflict of interest.
