# Supplementary figures and images for: Altered Light Sensitivity of Circadian Clock in Shank3+/– Mouse
Source: Front Neurosci. 2021 Feb 18;15:604165. doi: 10.3389/fnins.2021.604165 (PMC7930753; doi:10.3389/fnins.2021.604165)

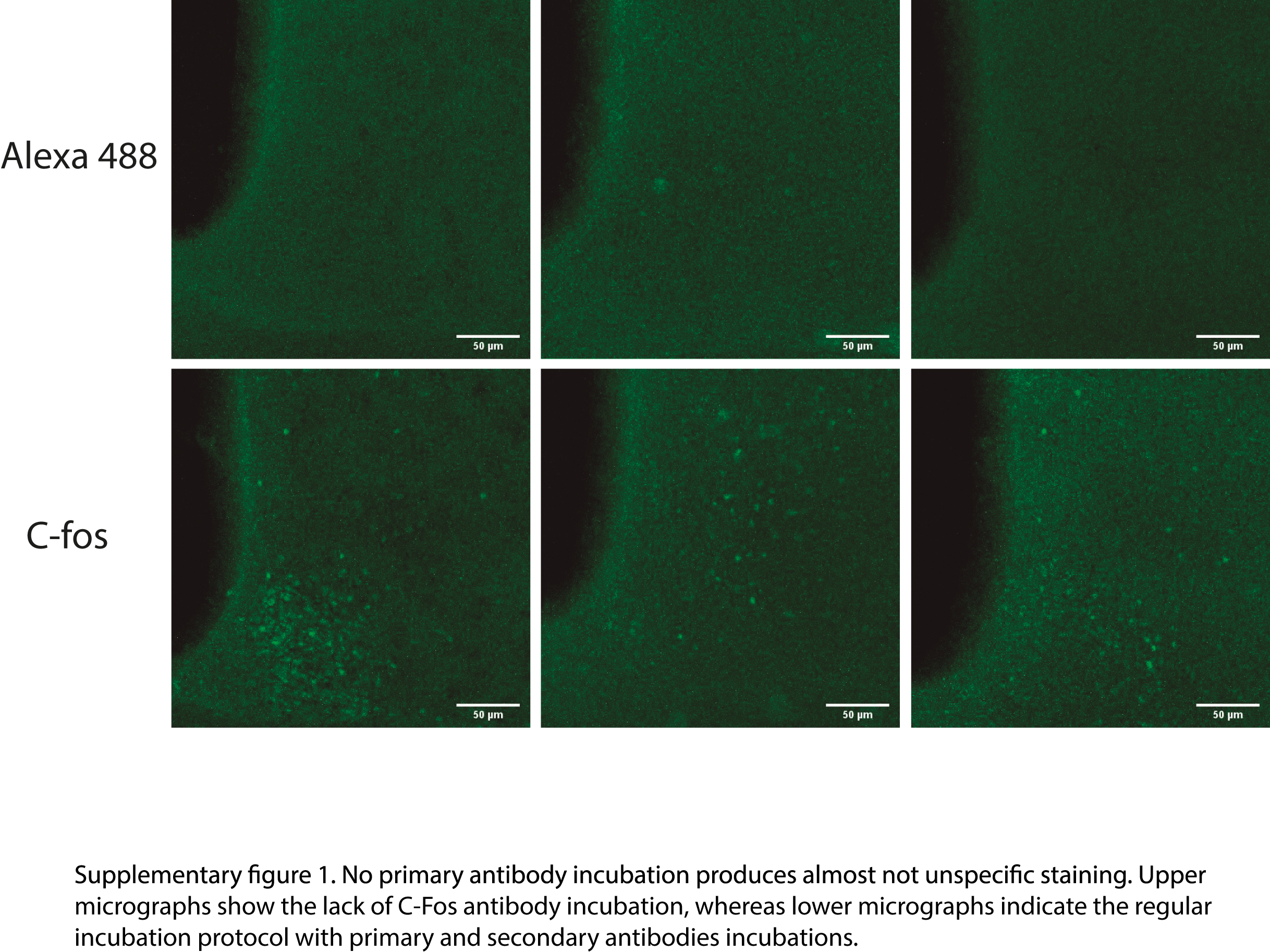

Supplement: Supplementary file 1 [file Image_1.JPEG]

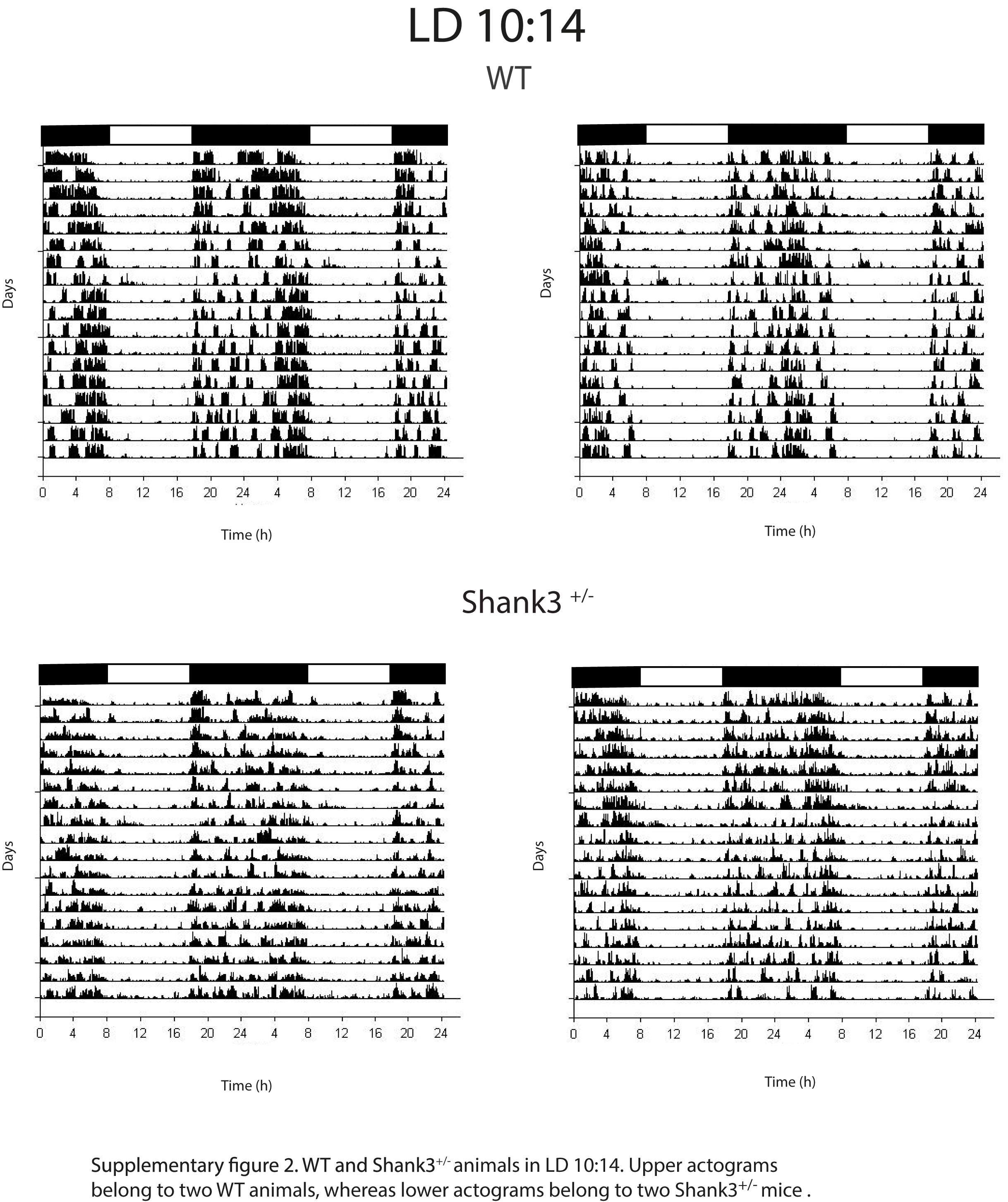

Supplement: Supplementary file 2 [file Image_2.JPEG]

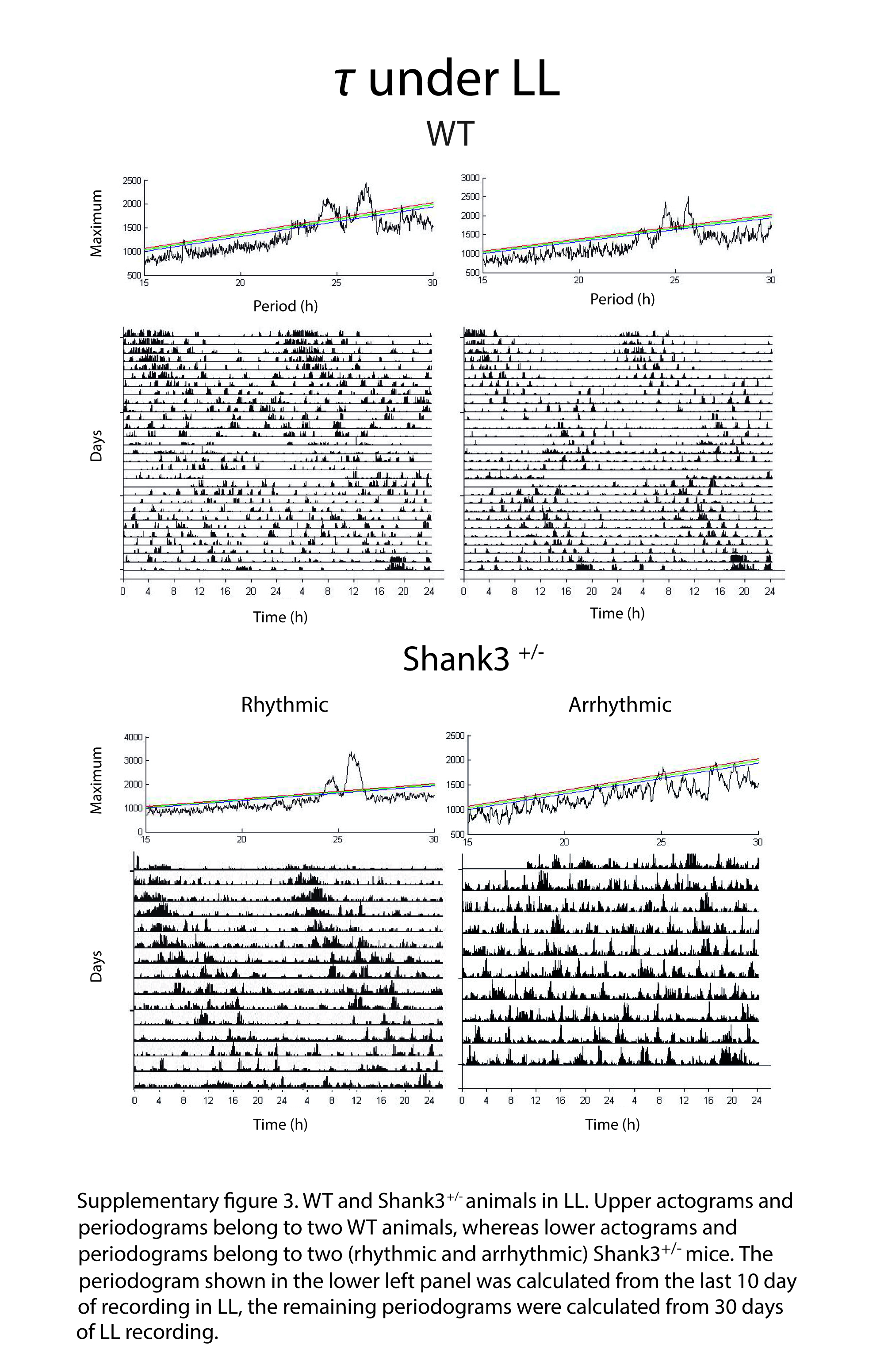

Supplement: Supplementary file 3 [file Image_3.jpg]

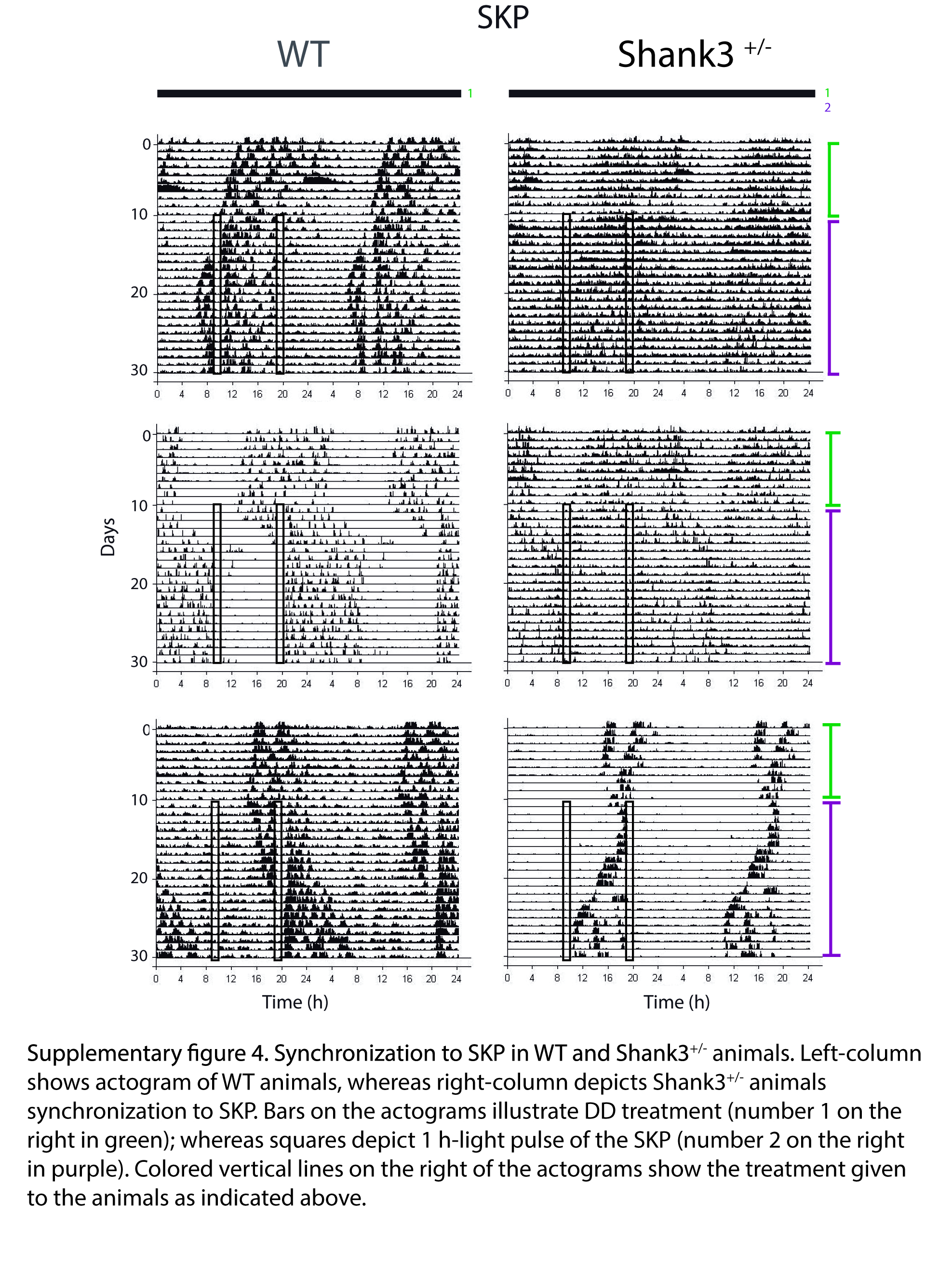

Supplement: Supplementary file 4 [file Image_4.JPEG]
